# Mapping the effect of the antisecretory factor on GABA_A_ receptor α_1_ and α_6_ subunits in cerebellar granule cells *in vitro*

**DOI:** 10.1016/j.ibneur.2024.08.001

**Published:** 2024-08-05

**Authors:** Virginia Bazzurro, Elena Gatta, Elena Angeli, Aroldo Cupello, Stefan Lange, Eva Jennische, Mauro Robello, Alberto Diaspro

**Affiliations:** a DIFILAB, Department of Physics, University of Genoa, Italy; b Department of Infectious Diseases, Institute of Biomedicine, University of Gothenburg, Sweden; c Region Västra Götaland, Department of Clinical Microbiology, Sahlgrenska University Hospital, Gothenburg, Sweden; d Department of Medical Biochemistry and Cell Biology, Institute of Biomedicine, Sahlgrenska Academy, University of Gothenburg, Sweden; e Nanoscopy, CHT Erzelli, Istituto Italiano di Tecnologia, Genoa, Italy

**Keywords:** Antisecretory factor, cerebellar granule cells, GABA_A_ receptors, immunofluorescence, 3D-STED microscopy, super-resolved microscopy

## Abstract

The Antisecretory Factor (AF) is a protein that can reduce intestinal hypersecretion and various inflammation disorders *in vivo*. Discovered in many mammalian tissues and plasma, its mechanism of action remains unknown. Interestingly, its induction has been found to counteract vertigo in patients with Méniere's disease. This suggests an inherent ability to control body balance and posture, an activity that may play a role in cerebellar function. Therefore, it may be worthwhile to investigate whether this activity can inhibit neuronal cells involved in cerebellar circuitries and its potential action on enteric nervous system ganglia, which could explain its antisecretory effect in the intestine.

Previously, we studied the role of AF on GABA_A_ receptors in cerebellar granule cells, taking advantage of electrophysiology and evaluating the effects of the administration of AF-16, an AF peptide. Treatment with AF-16 increased GABA_A_ receptor responses, especially those containing the α_6_ subunit. Here, we performed immunofluorescence experiments by staining α_1_ and α_6_ subunits before and after incubation with AF-16, analyzed super-resolved images comparing pre- and post-treatment maps and critically examined these experimental results with our previous electrophysiological data to shed light on the mechanisms of action of AF protein on GABA_A_ receptor subpopulations, specifically the "fast" receptors of α_n_ β_2/3_ γ_2_ composition that contain either the α_1_ or the α_6_ subunit.

The results indicate that the α_6_ subunit is redistributed, with a decrease in neurites and an increase in soma. Conversely, the α_1_ subunit shows opposite results, with an increase in neurites and a decrease in soma.

## Introduction

The antisecretory factor (AF) is a 41 kDa protein discovered, purified, and cloned in the ‘80 s by Lange and Lönnroth (Lange and Lönnroth, 2001).

AF works by preventing fluid hypersecretion caused by enteric toxins (Lönnroth and Lange, 1984, Lönnroth and Lange, 1985, Lange and Lönnroth, 1986, Torres et al., 1991) and other pathological disorders (Eriksson et al., 2003a, Eriksson et al., 2003b, Hanner et al., 2004, Hanner et al., 2010, Gatzinsky et al., 2020). It exerts its antisecretory effect through the small amino acidic region (I)VCHSKTR between residues 35 and 42 (Johansson et al., 1997).

Although AF can be found in many tissues and organs - respiratory organs, gastrointestinal tract and gallbladder, kidneys, ureters and urinary bladder, pituitary gland, and central nervous system (Lange and Lönnroth, 2001) - its mechanism of action remains unclear.

The study of AF is clinically relevant, the treatment with AF has improved the health of patients suffering from various pathological situations such as Ménière’s disease (Hanner et al., 2004, Hanner et al., 2010), Crohn's disease (Eriksson et al., 2003b), diarrheal diseases (Björck et al., 2000, Laurenius et al., 2003, Zaman et al., 2018), ulcerative colitis (Eriksson et al., 2003a), elevated intracranial pressure induced by traumatic brain edema (Gatzinsky et al., 2020) and glioma tumors (Kopecky et al., 2022, Ehinger et al., 2023).

These pathological conditions arise from an imbalance of ions and water transport across the plasma membrane. However, to gain a more detailed understanding of AF’s mechanism of action, it is necessary to determine its effect on cellular physiology through *in vitro* studies.

Studies have shown that AF functions through neuronal pathways (Lange et al., 1985, Lange et al., 1987, Rapallino et al., 1989, Rapallino et al., 2003), and its role in GABAergic transmission in the central nervous system (CNS) has been widely investigated in recent years (Kim et al., 2005, Strandberg et al., 2014, Bazzurro et al., 2018, Bazzurro et al., 2022). GABA is the principal inhibitory neurotransmitter in the mammalian CNS, acting via GABA_A_ and GABA_B_ receptors.

Our previous experiments using the patch-clamp technique demonstrated that the 16 amino acid AF-16 peptide (a fragment of the AF N-terminal end that contains the active site and is resistant to proteolytic degradation, Dzebo et al., 2014) affects GABA_A_ receptors expressed on the plasma membrane in cerebellar granule cells (Bazzurro et al., 2018, Bazzurro et al., 2022). After AF-16 treatment, the “peak” current induced by GABA increased. The effect was dependent on the concentration of the antisecretory factor and the incubation time, with the effect on GABA_A_ receptors rising as the concentration and the pretreatment time increased until reaching saturation (Bazzurro et al., 2018).

Previously, we investigated the action of AF-16 by electrophysiological experiments based on two-photon GABA uncaging technique (Cozzolino et al., 2020) and observed the different effects in various zones of interest (soma, axon initial segment, neurite). Using furosemide, an α_6_-containing GABA_A_ receptor antagonist, the results showed that the action of AF-16 in the neuron cell body is almost entirely blocked, suggesting the involvement of a specific GABA_A_ receptor population composed of α_6_ subunit (Bazzurro et al., 2022). These results are interesting, especially when considering the significant impact of AF production stimulation in the body, for instance, the decrease of vertigo in Meniere’s syndrome patients (Hanner et al., 2004, Hanner et al., 2010). Cerebellar granule cells are part of a circuitry that controls balance and posture by innervating cerebellar Purkinje cells. These cells, in turn, control the neurons of the lateral vestibular nucleus via a GABA-mediated input (Ito et al., 1966, Ito, 1984, Hydén et al., 2000).

This neuronal model can help understand the potential mechanisms underlying AF's role in the nervous system. It can also provide insights into the possibility that action on GABA_A_ receptors of the ENS might mediate its antisecretory activity in the intestine (Auteri et al., 2015, Poulter et al., 1999, Seifi et al., 2014, Seifi and Swinny, 2019).

Thus, in this study, we aimed to investigate the action of AF-16 using optical microscopy and to correlate it with the results obtained with electrophysiology. Both non-permeabilized and permeabilized cells were investigated to distinguish membrane-associated GABA_A_ receptors from those in the cell’s cytoplasm.

We focused on the role of the protein AF on GABA_A_ receptors and evaluated the variations of fluorescence intensity in control and AF-16 treated samples.

We estimated the fluorescence by measuring the fluorescence integrated density of α_6_ and α_1_ containing GABA_A_ receptors by comparing, 1-hour AF-16 pretreatment, samples with control untreated cells. We used indirect immunostaining and tridimensional-stimulated emission depletion (3D-STED)-based super-resolution microscopy to assess the fluorescence.

We compared the fluorescence integrated density of α_6_ and α_1_-containing GABA_A_ receptors in 1-hour AF-16 pretreated samples with control untreated cells. Our data showed a different behavior of α_6_ and α_1_ containing GABA_A_ receptors in the neurons' soma and neurites. The α_6_ GABA_A_ subtypes exhibited an increase in fluorescence, after AF incubation, in the cell body membranes and a reduction in neurites’ membranes. In contrast, the α_1_ GABA_A_ subtypes presented a decreased intensity in the soma and increase of fluorescence in the neuron neurites’ cytoplasm compared to the control neurons.

These results obtained through fluorescence methods confirmed our previous assumption, tested with electrophysiology, that AF acts on a specific population of GABA_A_ receptors. In turn, this suggests ideas about how the factor can influence cerebellar function and prevent vertigo in Méniere’s disease, and the possibility of an action on neurons in gastrointestinal tract ganglia.

## Materials and methods

### AF-16 peptide production

The AF-16 peptide composed of antisecretory factor (AF) amino acid sequences 36–51 (VCHSKTRSNPENNVGL) was synthesized with solid-phase synthesis by Ross-Petersen AS (Copenhagen, Denmark), as reported by Rapallino et al. (2003). AF-16 has been used as an adequate molecule to study the activity of the whole-size AF, since it comprises the active site and is resistant to proteolytic degradation (Dzebo et al., 2014).

### Animals

The Department of Pharmacy, Section of Pharmacology and Toxicology of the University of Genoa, housed Sprague-Dawley rats. These animals were treated in accordance with the E.U. Parliament and Council Directive of September 22nd, 2010 (2010/63/E.U.). The Italian Ministry of Health approved their use (COD. 75F11.N.6DX) D.M. 116/1992. All necessary measures were taken to minimize animal suffering and reduce the number of animals used.

### Cerebellar granule cell primary cultures

Cerebellar granule cells (CGC) were obtained from male and female 6–8 day old Sprague-Dawley rats using methods previously described (Robello et al., 1993).

The cells were plated at a density of 4.2 ×10^5^ per 13 mm poly-L-lysine-coated glass coverslips and cultured in a humidified 95 % air 5 % CO_2_ atmosphere at 37°C in a Basal Medium Eagle supplemented with 10 % fetal calf serum (Sigma-Aldrich, St. Louis, MO, USA), 25 mM KCl, 2.0 mM glutamine, 100 µg/ml gentamicin.

To prevent glial cell proliferation, 10 μM cytosine arabinoside (Sigma-Aldrich, St. Louis, MO, USA) was added to the culture medium 18–24 hours after plating, and the medium was refreshed after 48 hours with the addition of 10 μM cytosine arabinoside.

The experiments were conducted on the 6th-10th day *in vitro*.

### Immunolabeling and 3D-STED microscopy

For the immunolabeling experiments, control cells and cells that were incubated with 1.0 µM AF-16 for 1 hour at 37 °C were used.

The neurons were fixed in 4 % paraformaldehyde in PBS (Sigma-Aldrich, St. Louis, MO, USA) for 15 minutes at room temperature and washed three times with PBS. Some control and AF-16 pretreated samples were permeabilized with 0.1 % Triton X-100 (Sigma-Aldrich, St. Louis, MO, USA) in PBS for 5 minutes. All the samples were then blocked with 2 % BSA (bovine serum albumin) in PBS for 30 minutes to prevent non-specific binding and were incubated overnight at 4 °C with primary antibodies.

The primary antibodies used were as follows:

- Polyclonal rabbit antibody against rat GABA_A_ receptor α_6_ subunit (Alomone Labs Ltd., Israel) at a concentration of 1:500 in 2 % BSA;
- Polyclonal rabbit antibody against rat GABA_A_ receptor α_1_ subunit (Alomone Labs Ltd., Israel) at a concentration of 1:500 in 2 % BSA.
  The cells were washed three times with PBS and incubated with secondary antibodies for 45 minutes at room temperature.
  The secondary antibodies used were:
- ATTO594 anti-rabbit IgG against α_6_ subunit (Sigma-Aldrich, St. Louis, MO, USA) at a concentration of 1:200 in 2 % BSA;
- Abberior STAR580 anti-rabbit IgG against α_1_ subunit (Abberior GmbH, Göttingen, Germany) at a concentration of 1:200 in 2 % BSA.

The samples were washed three times with PBS and mounted in Mowiol® (Sigma-Aldrich, St. Louis, MO, USA).

The images were captured using a Stellaris 8 Falcon TauSTED (Leica Microsystems, Mannheim, Germany) inverted microscope. A white light laser provided the excitation wavelengths with a notch filter set at 561 nm and a STED beam laser with a wavelength of 775 nm. An avalanche photodiode was used as a detector in the 571–700 nm range. The samples were imaged through a plan-apochromatic oil immersion objective 100×/1.40 NA. 3D Z-stacks (512 × 512 pixels, 16- bit) were acquired with a Z-step size of 0.07 µm, setting the pinhole at 1.0 Airy size, speed of 200 Hz, and field of view of 47.68 μm × 47.68 μm.

### Image processing and statistical analysis

**Fig. 1:**
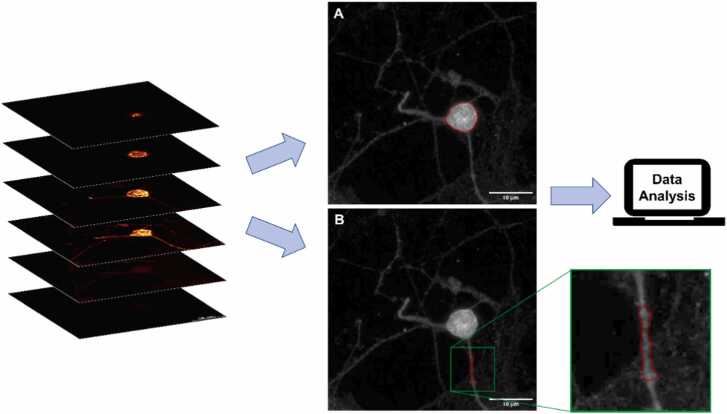
Example of a Z-stack analyzed using the sorftware Fiji. After evaluating the volume of the neuron, we projected the sum of the fluorescence intensity of the 3D frames on the Z-axis. We subtracted the fluorescence noise background and drew a region of interest (ROI) around the soma (A) or neurite (B). The obtained values of the ROI integrated density were normalized with the cell volume.

Z-stacks were analyzed using the software Fiji. We evaluated the sum of the fluorescence intensity of each single frame of the 3D stack using the Z-projection function. After subtracting the image background, we determined the fluorescence intensity by drawing a region of interest (ROI) around every cell's profile (soma or neurite) (see Fig. 1).

The obtained values of the ROI integrated density were normalized, taking into consideration the cell volume of each neuron. Then, we calculated the weighted average of the integrated density/volume ratio for every 3D acquisition.

Finally, we assessed the effect of AF-16 by calculating the percentage effect with the following formula:

$$E\%=\left(\frac{I_{\mathrm{AF}-16}-I_{\mathrm{CTRL}}}{I_{\mathrm{CTRL}}}\right)∙100$$

where I_AF-16_ is the treated cell fluorescence integrated density, and I_CTRL_ is the control neuron fluorescence integrated density.

The values used in the final calculations (I_AF-16_ and I_CTRL_) were the means across different evaluations in the various sets of cells according to the treatment (AF-16 or controls), permeabilization or not-permeabilization, and α_6_ or α_1_ subunit, cell soma or neurites: overall, sixteen different sets. To assess the statistical significance of E% comparisons in the various instances, the E% (see formula above) SEM’s were evaluated as described in the following paragraph.

Data are reported as mean ± SEM. Error propagation was used to calculate SEM for E% in all comparisons with the controls. In the comparison of the various pairs of E% values, we employed the Student's t-test. Statistical significance was set at p < 0.05.

## Results

We used a concentration of 1.0 μM AF-16 to test the effect of AF on cerebellar granule cells. This concentration allowed us to work with the lowest concentration, while still having the maximum effect as demonstrated in our previous work's dose-response curve (Bazzurro et al., 2018).

We then used immunofluorescence microscopy approaches to investigate the impact of AF-16 on two different subpopulations of GABA_A_ receptors, α_1_ and α_6_-containing GABA_A_Rs, in both non-permeabilized and permeabilized cerebellar granule cultures.

The results of the immunofluorescence tests showed a different behavior for α_1_ and α_6_-containing GABA_A_ receptors.

**Fig. 2:**
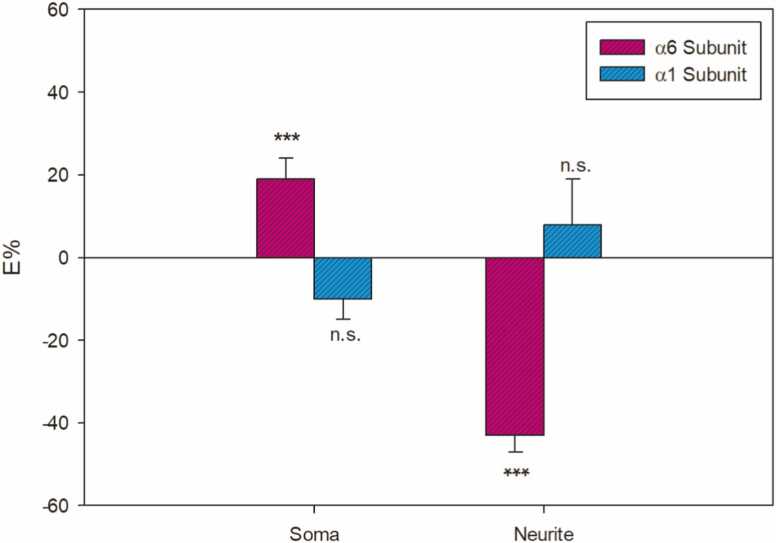
1.0 µM AF-16 percentage effect for α_6_- (magenta) and α_1_-containing GABA_A_Rs (blue) in non-permeabilized granule cell cultures. Vertical bars represent SEMs. Student’s t-test evaluated E% statistical significance: ***p < 0.001, n.s. is not significant.

In non-permeabilized cell cultures, the α_6_ subunit's fluorescence integrated density significantly (p < 0.001) increased by (19±5)%, whereas the α_1_-containing GABA_A_Rs showed only a tendency to decrease by (10±5)% (n.s.) in the neuronal soma. In non-permeabilized cell neurites, the α_6_ GABA_A_Rs decreased (p < 0.001) by (43±4)%, while the α_1_ subunit did not change (8±11, n.s.)% (Fig. 2).

We also carried out experiments in samples permeabilized with 0.1 % Triton X-100 to compare the results with the non-permeabilized ones.

In the cell body of permeabilized samples, AF-16 treatment led to a significant (p < 0.01) decrease of α_1_ by (-16±1)%. Here, the α_6_ subunit increase of (23±2)% did not reach statistical significance.

**Fig. 3:**
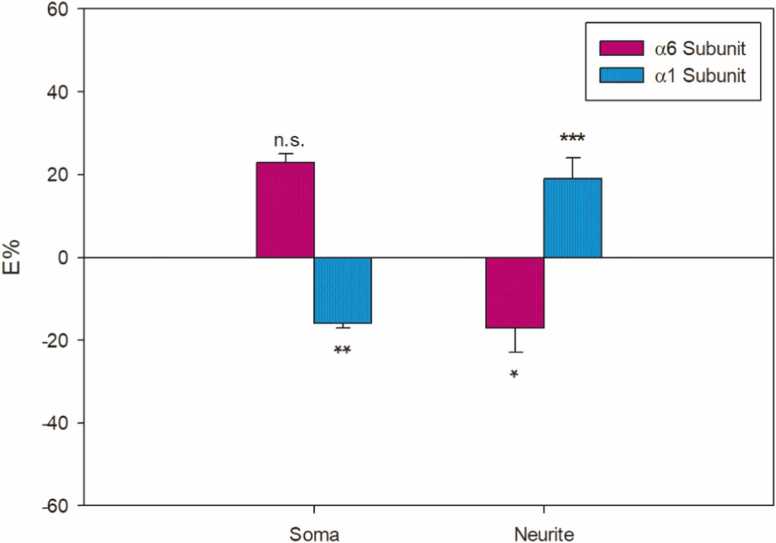
1.0 µM AF-16 percentage effect for α_6_- (magenta) and α_1_-containing GABA_A_Rs (blue) in permeabilized granule cell cultures. Vertical bars represent SEMs. Student’s t-test evaluated E% statistical significance: *p < 0.05, **p < 0.01, ***p < 0.001, n.s. is not significant.

In the permeabilized cell neurites, the opposite occurred: the α_6_ subunit significantly (p < 0.05) decreased by (-17±6)%, while the α_1_ subunit increased (p < 0.001) by (19±5)% (Fig. 3).

Table 1, Table 2 summarize the results presented as histograms in Fig. 2, Fig. 3. Table 3 shows that in all comparisons (either for non-permeabilized or permeabilized cells; either α_6_ or α_1_ subunit) between soma and neurites, the effect (E%) of AF-16 was different. In particular, the effect on the subunit density for α_6_ was an increase in the soma statistically different from the (decreased) value in the neurites, both for permeabilized (referring to cytoplasm plus plasma membranes) and non-permeabilized (referring only to the plasma membranes) CGC neurons. The contrary applies to the α_1_ subunit. Overall, this demonstrates an opposite trafficking of the two subunits, induced by the action of AF-16. From neurites to soma for α_6_ subunits, vice versa for the α_1_ ones.

**Table 1:** Percentage effect (E%) of 1.0 μM AF-16 in non-permeabilized samples of cerebellar granule cultures. E% are given as mean ± SEM. P-value was evaluated with Student’s t-test; n.s. is not significant. n_CTRL_ is the number of control cells, and n_AF-16_ is the number of treated neurons.

**Non-permeabilized samples**	**E% ± SEM**	**Statistical significance**	**n**
**α**_**6**_**GABA**_**A**_**Rs Soma**	19 ± 5	p < 0.001	n_CTRL_ = 68; n_AF−16_ = 59
**α**_**6**_**GABA**_**A**_**Rs Neurite**	−43 ± 4	p < 0.001	n_CTRL_ = 66; n_AF−16_ = 56
**α**_**1**_**GABA**_**A**_**Rs Soma**	−10 ± 5	n.s.	n_CTRL_ = 64; n_AF−16_ = 73
**α**_**1**_**GABA**_**A**_**Rs Neurite**	8 ± 11	n.s.	n_CTRL_ = 43; n_AF−16_ = 43

**Table 2:** Percentage effect (E%) of 1.0 μM AF-16 in permeabilized samples of cerebellar granule cultures. E% are given as mean ± SEM. The p-value was evaluated with Student’s t-test; n.s. is not significant. n_CTRL_ is the number of control cells, and n_AF-16_ is the number of treated neurons.

**Permeabilized samples**	**E% ± SEM**	**Statistical significance**	**n**
**α**_**6**_**GABA**_**A**_**Rs Soma**	23 ± 2	n.s.	n_CTRL_ = 112; n_AF−16_ = 105
**α**_**6**_**GABA**_**A**_**Rs Neurite**	−17 ± 6	p < 0.05	n_CTRL_ = 51; n_AF−16_ = 48
**α**_**1**_**GABA**_**A**_**Rs Soma**	−16 ± 1	p < 0.01	n_CTRL_ = 175; n_AF−16_ = 156
**α**_**1**_**GABA**_**A**_**Rs Neurite**	19 ± 5	p < 0.001	n_CTRL_ = 61; n_AF−16_ = 47

**Table 3:** Statistical significance, calculated with Student’s t-test, of the results for soma-neurite in non-permeabilized and permeabilized samples for α_1_ and α_6_-containing GABA_A_Rs.

	**Statistical significance**
**Non-permeabilized α**_**6**_**GABA**_**A**_**Rs Soma-Neurite**	p < 0.001
**Non-permeabilized α**_**1**_**GABA**_**A**_**Rs Soma-Neurite**	p < 0.01
**Permeabilized α**_**6**_**GABA**_**A**_**Rs Soma-Neurite**	p < 0.05
**Permeabilized α**_**1**_**GABA**_**A**_**Rs Soma-Neurite**	p < 0.05

Table 4 shows the comparison of cells cytoplasm plus plasma membranes with only plasma membrane subunit density in soma or neurites separately. Only for α_6_ and in neurites, the subunit density change was different in non-permeabilized (only plasma membranes) *vs*. permeabilized (cytoplasm plus plasma membranes) neurons. The results show that for α_6,_ its decrease in plasma membranes is significantly greater than in cell cytoplasm.

**Table 4:** Statistical significance, calculated with Student’s t-test, for non-permeabilized and permeabilized samples either for α_1_ and α_6_ subunits in soma or neurite. n.s. is not significant.

	**Statistical significance**
**Non-permeabilized-permeabilized α**_**6**_**GABA**_**A**_**Rs Soma**	n.s.
**Non-permeabilized-permeabilized α**_**1**_**GABA**_**A**_**Rs Soma**	n.s.
**Non-permeabilized-permeabilized α**_**6**_**GABA**_**A**_**Rs Neurite**	p < 0.01
**Non-permeabilized-permeabilized α**_**1**_**GABA**_**A**_**Rs Neurite**	n.s.

**Fig. 4:**
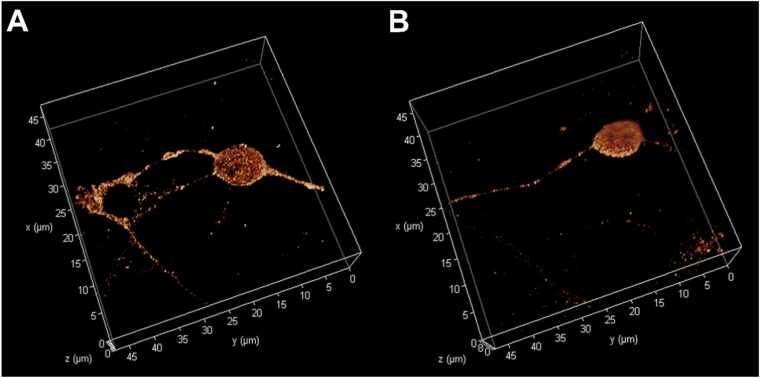
3D-STED reconstruction of permeabilized cerebellar granule cells labeled with primary antibodies anti-α_6_ GABA_A_ receptor and ATTO594 secondary antibodies. The Z-stacks were acquired at 512 × 512 pixels, 47.68 μm × 47.68 μm, 16-bit, 200 Hz, 2-line averages, 0.070 μm Z-step size, 1.0 Airy disk. A shows the control samples; B shows 1 h treated samples with 1.0 μM AF-16.

**Fig. 5:**
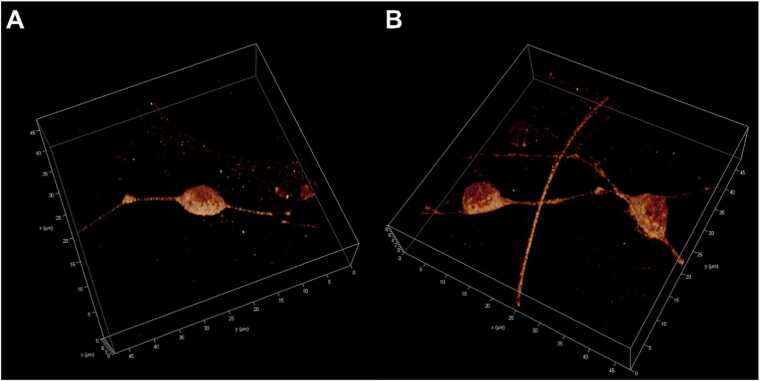
3D-STED reconstruction of permeabilized cerebellar granule cells labeled with primary antibodies anti-α_1_ GABA_A_ receptor and Abberior STAR580 secondary antibodies. The Z-stacks were acquired at 512 × 512 pixels, 47.68 μm × 47.68 μm, 16-bit, 200 Hz, 2-line averages, 0.070 μm Z-step size, 1.0 Airy disk. A shows the control samples; B shows 1 h treated samples with 1.0 μM AF-16.

We included 3D reconstructions of cerebellar granule cells labeled with primary antibodies anti-α_6_ or anti-α_1_ GABA_A_Rs’ subunits in permeabilized CGC cells, which are shown in Fig. 4, Fig. 5, respectively.

## Discussion

The Antisecretory Factor was discovered by Lönnroth and Lange in 1985. Since then, it has been characterized, cloned, and sequenced (Johansson et al., 1995) and has been found to be present in several organs, in addition to the pituitary and intestine (Lange et al., 1999). Originally, it was indicated and studied as an endogenous protein that could counteract intestinal hypersecretion (Lönnroth and Lange, 1985, Lange and Lönnroth, 2001) and has been successfully used in clinical settings to counteract pathologies resulting in diarrhea (Björck et al., 2000; Eriksson et al., 2003a; Eriksson et al., 2003b; Laurenius et al., 2003; Zaman et al., 2018). The idea that it could also play a role in regulating water in body fluids led to trials in pathologies such as Méniere’s disease, where hypersecretion of endolymph in the inner ear is thought to be the cause of the disease (Gibson, 2010, Brown et al., 2013, Mohseni-Dargah et al., 2023). In these clinical studies, the major therapeutic result was a decrease in vertigo symptoms (Hanner et al., 2004, Hanner et al., 2010). However, a possible mechanism of action of AF via the nervous system has been proposed (Lange and Lönnroth, 2001, Grøndahl et al., 2002). For such reasons, studies have been conducted to investigate the interaction of AF with the physiology of brain nervous system structures (Kim et al., 2005, Strandberg et al., 2014). In addition to searching for specific functions in the CNS of the factor AF, it is important to understand whether its activity in counteracting hypersecretion in the intestine is via enteric nervous system ganglia. When considering the beneficial effect against vertigo, it is appropriate to consider actions in terms of cerebellar circuitries, which contribute to body posture and balance control. The circuit mossy fibers-cerebellar granule cells (under inhibitory control by cerebellar Golgi cells)-Purkinje cells-deep cerebellar nuclei-lateral vestibular nucleus Deiters’ neurons-spinal cord anti-gravity motor neurons (Ito et al., 1966, Ito, 1984, Hydén et al., 2000) certainly has a role in this connection. The model of primary rat cerebellar granule cells culture has been long used by our group for studying their inhibitory GABA_A_ system (Robello et al., 1993, Gatta et al., 2009, Cupello et al., 2013). Thus, we conducted a study to investigate the effects of AF-16 on the inhibitory GABA-mediated chloride currents in CGCs in culture. The results clearly showed that 1.0 µM AF-16 caused an increase (by 40 %) of GABA_A_ receptor-mediated “peak” chloride currents (Bazzurro et al., 2018). This current component (Robello et al., 1999) is due to GABA_A_ receptors of specific subunit compositions: α_6_β_2/3_γ_2_, α_1_β_2/3_γ_2_ and α_1_α_6_β_2/3_γ_2_ (Gatta et al., 2009). It corresponds to the “fast” synaptic inhibition of cerebellar granule cells *in situ* (Farrant and Nusser, 2005), whereas the “steady state” component (Robello et al., 1999) corresponds to the “tonic” inhibition *in situ* (Farrant and Nusser, 2005). Later, we demonstrated that GABA_A_ receptors containing the α_6_ subunit played a major role in that increase (Bazzurro et al., 2022). Our present results indicate a redistribution of GABA_A_ receptors containing the α_6_ subunit in CGC with an increased presence in the cells’ body *vs.* that in neurites. We found also the opposite change of those containing the α_1_ subunit. Another interesting result (Table 4) is that in neurites the plasma membrane pool of α_6_-containing receptors undergoes a greater decrease than the cytoplasmic one. This happens in correspondence with its significant increase in somatic membranes (Fig. 2).

Considering the different functional profile of α_6_β_2/3_γ_2_ and α_1_β_2/3_γ_2_ GABA_A_ receptors both in terms of affinity for GABA (Sigel and Baur, 2000) and deactivation/desensitization (Tia et al., 1996), this redistribution on plasma membranes can bring about a different efficiency of the inhibitory control on such neurons. On the other hand, our present results cannot exclude that the α_6_ subunit relative overall increase in the cell body is related to “tonic” δ subunit containing receptors (Farrant and Nusser, 2005) which are extra-synaptic and mainly present in cerebellar granules cell body (Merlo et al., 2004). This seems to be denied by the absence of changes in the “steady state” current component in CGC’s in culture (Bazzurro et al., 2018). However, subtler differences in α_6_β_2/3_δ and α_1_β_2/3_δ. GABA_A_ receptors (*e.g.,* in desensitization kinetics or affinity for GABA, Sieghart et al., 2022) may have a role, in absence of changes in the current intensity as found in our model. Within cerebellar circuitries, changes in the CGC inhibition processes may be factors in the anti-vertigo action of AF. Recent results show in a mouse model of Essential Tremor (ET), a neurological disorder, which probably involves synchronous rhythmic firing of cerebellar Purkinje cells, a protective effect by drugs which positively modulate cerebellar granule cell GABA_A_ receptors containing the α_6_ subunit. Among these, ethanol at low levels and pyrazoloquinolinones (Huang et al., 2023*;* Sieghart et al., 2022). Previous work had indicated in the cerebellar granule cells α_6_βδ receptors those whose activation protects from Essential Tremor (Handforth et al., 2018).

The explanation of the protective effect on the ET model by drugs positively activating α_6_ subunit containing receptors was a re-equilibration of a deranged activity of the Inferior Olive Nucleus (ION)-Purkinje cells (PC)-Deep Cerebellar Nuclei (DCN)-Inferior Olive Nucleus (ION) circuit. This was suggested to happen via increased inhibition of cerebellar granule cells and a resulting less active excitatory input by these cells on Purkinje cells (Huang et al., 2023). Something similar could happen by the enhancing action of AF on inhibition of cerebellar granule cells in the circuit involving mossy fibers-cerebellar granule cells-Purkinje cells-deep cerebellar nuclei-vestibular neurons-mossy fibers originating in the nucleus reticularis tegmenti pontis (Fanardjian and Sarkisian, 1988).

It has to be established whether changes in GABA_A_ receptors composition and distribution, involving the α_6_ subunit, may play a role in the intestinal antisecretory activity of AF, possibly via an action on ENS ganglia. The presence of α_6_ subunit in ENS ganglia is only marginal (Poulter et al., 1999). However, this subunit has been found in neurons in the human and pig small intestine Meissner’s plexus and human Auerbach plexus (J*ennische and Lange, 2021*, unpublished results). Thus, it cannot be excluded a mechanism of counteraction of intestinal hypersecretion via ENS, although it remains to be demonstrated.

Summing up, our present experiments and results support the idea that AF can interfere with cerebellar mechanisms, possibly with those related to body posture and balance, via modification of the inhibitory input to cerebellar granule cells. Whether changes in the function of GABA_A_ receptors of ENS ganglia play a role in its antisecretory action in the intestine has yet to be demonstrated.

## Compliance with ethical standards

Please note that all authors have read and have abided by the statement of ethical standards for manuscripts submitted to IBRO Neuroscience Reports. All authors have approved the final article.

## CRediT authorship contribution statement

**Stefan Lange:** Writing – original draft, Supervision. **Aroldo Cupello:** Writing – original draft, Data curation, Conceptualization. **Alberto Diaspro:** Writing – original draft, Supervision, Funding acquisition. **Mauro Robello:** Writing – original draft, Supervision. **Eva Jennische:** Writing – original draft, Supervision. **Elena Gatta:** Writing – original draft, Supervision, Methodology, Investigation, Data curation, Conceptualization. **Virginia Bazzurro:** Writing – review & editing, Writing – original draft, Investigation, Formal analysis, Data curation. **Elena Angeli:** Writing – original draft, Supervision, Conceptualization.

## Declaration of Competing Interest

The authors declare that they have no known competing financial interests or personal relationships that could have appeared to influence the work reported in this paper.

## References

[bib1] Auteri M., Zizzo M.G., Serio R. (2015). GABA and GABA receptors in the gastrointestinal tract: from motility to inflammation. Pharmacol. Res..

[bib2] Bazzurro V., Gatta E., Angeli E., Cupello A., Lange S., Jennische E., Robello M., Diaspro A. (2022). Involvement of GABA_A_ receptors containing α_6_ subtypes in antisecretory factor activity on rat cerebellar granule cells studied by two-photon uncaging. Eur. J. Neurosci..

[bib3] Bazzurro V., Gatta E., Cupello A., Lange S., Robello M. (2018). Antisecretory factor modulates GABA_A_ receptor activity in neurons. J. Mol. Neurosci..

[bib4] Björck S., Bosaeus I., Ek E., Jennische E., Lönnroth I., Johansson E., Lange S. (2000). Food induced stimulation of the antisecretory factor can improve symptoms in human inflammatory bowel disease: a study of a concept. Gut.

[bib5] Brown D.J., Chihara Y., Curthoys I.S., Wang Y., Bos M. (2013). Changes in cochlear function during acute endolymphatic hydrops development in guinea pigs. Hear Res..

[bib6] Cozzolino M., Bazzurro V., Gatta E., Bianchini P., Angeli E., Robello M., Diaspro A. (2020). Precise 3D modulation of electro-optical parameters during neurotransmitter uncaging experiments with neurons in vitro. Sci. Rep..

[bib7] Cupello A., Di Braccio M., Gatta E., Grossi G., Nikas P., Pellistri F., Robello M. (2013). GABA_A_ receptors of cerebellar granule cells in culture: interaction with benzodiazepines. Neurochem. Res..

[bib8] Dzebo M.M., Reymer A., Fant K., Lincoln P., Nordén B., Rocha S. (2014). Enhanced cellular uptake of antisecretory peptide AF-16 through proteoglycan binding. Biochemistry.

[bib9] Ehinger E., Kopecky J., Darabi A., Visse E., Edvardsson C., Tomasevic G., Cederberg D., Belting M., Bengzon J., Siesjö P. (2023). Antisecretory factor is safe to use as add-on treatment in newly diagnosed glioblastoma. BMC Neurol..

[bib10] Eriksson A., Shafazand M., Jennische E., Lange S. (2003). Effect of antisecretory factor in ulcerative colitis on histological and laborative outcome: a short period clinical trial. Scand. J. Gastroenterol..

[bib11] Eriksson A., Shafazand M., Jennische E., Lönnroth I., Lange S. (2003). Antisecretory factor-induced regression of Crohn’s disease in a weak responder to conventional pharmacological treatment. Inflamm. Bowel Dis..

[bib12] Fanardjian V.V., Sarkisian V.A. (1988). Synaptic mechanisms of interaction of lateral vestibulo-spinal neurons with some brainstem structures. Prog. Brain Res*.*.

[bib13] Farrant M., Nusser Z. (2005). Variations on an inhibitory theme: phasic and tonic activation of GABA_A_ receptors. Nat. Rev. Neurosci..

[bib14] Gatta E., Cupello A., Pellistri F., Robello M. (2009). GABA_A_ receptors of cerebellar granule cells in culture: explanation of overall insensitivity to ethanol. Neuroscience.

[bib15] Gatzinsky K., Johansson E., Jennische E., Oshalim M., Lange S. (2020). Elevated intracranial pressure after head trauma can be suppressed by antisecretory factor - a pilot study. Acta Neurochir..

[bib16] Gibson W.P.R. (2010). Hypothetical mechanism for vertigo in Méniere’s disease. Otolaryngol. Clin. N. Am..

[bib17] Grøndahl M.L., Sørensen H., Unmack M.A., Holm A., Skadhauge E. (2002). Neuronal involvement in the effect of an antisecretory factor-derived peptide on induced secretion in the porcine small intestine. J. Comp. Physiol. A.

[bib18] Handforth A., Kadam P.A., Kosoyan H.P., Eslami P. (2018). Suppression of harmaline tremor by activation of an extrasynaptic GABAA receptor: implications for essential tremor. Tremor Other Hyperkinetic Mov..

[bib19] Hanner P., Jennische E., Lange S., Lönnroth I., Wahlström B. (2004). Increased antisecretory factor reduces vertigo in patients with Ménière’s disease: a pilot study. Hear Res..

[bib20] Hanner P., Rask-Andersen H., Lange S., Jennische E. (2010). Antisecretory factor-inducing therapy improves the clinical outcome in patients with Ménière’s disease. Acta Otolaryngol..

[bib21] Huang Y.-H., Lee M.T., Hsueh H.-Y., Knutson D.E., Cook J., Mihovilovic M.D., Sieghart W., Chiou L.-C. (2023). Cerebellar α6GABA_A_ receptors as a therapeutic target for essential tremor: proof‑of‑concept study with ethanol and pyrazoloquinolinones. Neurotherapeutics.

[bib22] Hydén H., Rapallino M.V., Cupello A. (2000). Unraveling of important neurobiological mechanisms by the use of pure, fully differentiated neurons obtained from adult animals. Progr. Neurobiol..

[bib23] Ito M. (1984).

[bib24] Ito M., Obata K., Ochi R. (1966). The origin of cerebellar-induced inhibition of Deiters neurones. II. Temporal correlation between the trans-synaptic activation of Purkinje cells and the inhibition of Deiters neurons.. Exp. Brain Res..

[bib25] Johansson E., Lange S., Lönnroth I. (1997). Identification of an active site in the antisecretory factor protein. Biochim. Biophys. Acta.

[bib26] Johansson, Lönnroth I., Lange S., Jonson I., Jennische E., Lönnroth C. (1995). Molecular cloning and expression of a pituitary gland protein modulating intestinal fluid secretion. J. Biol. Chem..

[bib27] Kim M., Wasling P., Xiao M.-Y., Jennische E., Lange S., Hanse E. (2005). Antisecretory factor modulates GABAergic transmission in the rat hippocampus. Regul. Pept..

[bib28] Kopecky J., Pérez J.E., Eriksson H., Visse E., Siesjö P., Darabi A. (2022). Intratumoral administration of the antisecretory peptide AF16 cures murine gliomas and modulates macrophage functions. Sci. Rep..

[bib29] Lange S., Jennische E., Johansson E., Lönnroth I. (1999). The antisecretory factor: synthesis and intracellular localization in porcine tissues. Cell Tissue Res..

[bib30] Lange S., Lönnroth I., Palm A., Hydén H. (1985). An inhibitory protein of intestinal fluid secretion reverses neuronal GABA transport. Biochem. Biophys. Res. Commun..

[bib31] Lange S., Lönnroth I. (1986). Bile and milk from cholera toxin treated rats contain a hormone-like factor which inhibits diarrhea induced by the toxin. Int. Arch. Allergy Appl. Immunol..

[bib32] Lange S., Lönnroth I. (2001). The antisecretory factor: synthesis, anatomical and cellular distribution, and biological action in experimental and clinical studies. Int. Rev. Cytol..

[bib33] Lange S., Lönnroth I., Palm A., Hydén H. (1987). The effect of antisecretory factor on the permeability of nerve cell membrane to chloride ion. Pflug. Arch..

[bib34] Laurenius A., Wängberg B., Lange S., Jennische E., Lundgren B.K., Bosaeus I. (2003). Antisecretory factor counteracts secretory diarrhoea of endocrine origin. Clin. Nutr..

[bib35] Lönnroth I., Lange S. (1984). Inhibition of cyclic AMP-mediated intestinal hypersecretion by pituitary extracts from rats pretreated with cholera toxin. Med. Biol..

[bib36] Lönnroth I., Lange S. (1985). A hormone-like protein from the pituitary gland inhibits intestinal hypersecretion induced by cholera toxin. Regul. Pept. Suppl..

[bib37] Merlo F., Balduzzi R., Cupello A., Robello M. (2004). Immunocytochemical study by two photon fluorescence microscopy of the distribution of GABA_A_ receptor subunits in rat cerebellar granule cells in culture. Amino Acids.

[bib38] Mohseni-Dargah M., Falahati Z., Pastras C., Khajeh K., Mukherjee P., Razmjou A., Stefani S., Asadnia M. (2023). Méniere’s disease: pathogenesis, treatments and emerging approaches for an idiopathic bioenvironmental disorder.. Environ. Res..

[bib39] Poulter M.O., Singhal R., Brown L.A., Krantis A. (1999). GABA_A_ receptor subunit messenger RNA expression in the enteric nervous system of the rat: implications for functional diversity of enteric GABA_A_ receptors. Neuroscience.

[bib40] Rapallino M.V., Cupello A., Lange S., Lönnroth I., Hydén H. (1989). Further studies on the effect of ASF factor on Cl^-^ permeability across the Deiters' neurone plasma membrane. Int. J. Neurosci..

[bib41] Rapallino M.V., Cupello A., Lange S., Lönnroth I. (2003). Antisecretory factor peptide derivatives specifically inhibit [^3^H]-gamma-amino-butyric acid/^36^Cl^–^ out → in permeation across the isolated rabbit Deiters' neuronal membrane. Acta Physiol. Scand..

[bib42] Robello M., Amico C., Cupello A. (1993). Regulation of GABA_A_ receptor in cerebellar granule cells in culture: differential involvement of kinase activities. Neuroscience.

[bib43] Robello M., Amico C., Cupello A. (1999). Evidence of two populations of GABA_A_ receptors in cerebellar granule cells in culture: different desensitization kinetics, pharmacology, serine/threonine kinase sensitivity, and localization. Biochem. Biophys. Res. Commun..

[bib44] Seifi M., Brown J.F., Mills J., Bhandari P., Belelli D., Lambert J.J., Rudolph U., Swinny J.D. (2014). Molecular and functional diversity of GABA-A receptors in the enteric nervous system of the mouse colon. J. Neurosci..

[bib45] Seifi M., Swinny J.D. (2019). Developmental and age-dependent plasticity of GABA_A_ receptors in the mouse colon: implications in colonic motility and inflammation. Auton. Neurosci..

[bib46] Sieghart W., Chiou L.-C., Ernst M., Fabjan J., Savic M.M., Lee M.T. (2022). α6-containing GABA_A_ receptors: functional roles and therapeutic potentials. Pharmacol. Rev..

[bib47] Sigel E., Baur R. (2000). Electrophysiological evidence for the coexistence of α_1_ and α_6_ subunits in a single functional GABA_A_ receptor. J. Neurochem..

[bib48] Strandberg J., Lindquist C., Lange S., Asztely F., Hanse E. (2014). The endogenous peptide antisecretory factor promotes tonic GABAergic signaling in CA1 stratum radiatum interneurons. Front. Cell Neurosci..

[bib49] Tia S., Wang J.F., Kotchabhakdi N., Vicini S. (1996). Distinct deactivation and desensitization kinetics of recombinant GABA_A_ receptors. Neuropharmacology.

[bib50] Torres J., Jennische E., Lange S., Lönnroth I. (1991). *Clostridium difficile* toxin A induces a specific antisecretory factor which protects against intestinal mucosal damage. Gut.

[bib51] Zaman S., Aamir K., Hanson L.Å., Lange S. (2018). High doses of Antisecretory factor stop diarrhea fast without recurrence for six weeks post treatment. Int. J. Infect. Dis..

